# Spatial pharmacology of anti-cancer immunomodulators: a comprehensive review

**DOI:** 10.3389/fphar.2026.1898418

**Published:** 2026-08-12

**Authors:** Sixuan Chen, Liping Tu, Quanhua Chen

**Affiliations:** 1 Minxi Vocational and Technical College, Longyan, China; 2 Longyan People’s Hospital Affiliated to Xiamen Medical College, Longyan, China

**Keywords:** anti-cancer immunomodulators, cancer immunotherapy, intratumoral heterogeneity, spatial pharmacology, tumor microenvironment

## Abstract

The field of spatial pharmacology has emerged as a critical framework for understanding the complex interactions between anti-cancer immunomodulators, tumor cells, and the tumor microenvironment (TME) at single-cell resolution. This review synthesizes the theoretical foundations, historical evolution, clinical applications, diagnostic techniques, therapeutic strategies, and future directions of spatial pharmacology in cancer immunotherapy. By integrating single-cell analysis, signaling dynamics, and multimodal approaches, spatial pharmacology addresses the limitations of traditional “one-size-fits-all” therapies by accounting for intratumoral heterogeneity, drug target accessibility, and immune cell spatial organization. Key advancements include single-cell RNA sequencing (scRNA-seq) for mapping drug target pathway activation, imaging techniques for visualizing signaling dynamics, and multimodal diagnostics for personalized treatment design. Clinical applications range from optimizing combinatorial regimens in metastatic renal cell carcinoma to predicting immunotherapy response in triple-negative breast cancer. Despite challenges such as overcoming drug resistance and enhancing target accessibility, emerging technologies like spatial transcriptomics and artificial intelligence (AI)-driven multi-omics integration promise to revolutionize precision oncology. This review highlights how spatial pharmacology is transforming cancer immunotherapy by enabling tailored interventions that account for the spatial and temporal complexity of tumor-immune interactions.

## Spatial pharmacology of anti-cancer immunomodulators: theoretical foundations

### Single-cell heterogeneity in drug target accessibility

Intratumoral heterogeneity remains a major barrier to effective cancer treatment, as targeted therapies often eliminate only specific subpopulations of tumor cells while leaving others unharmed (Kim et al., 2016). Single-cell RNA sequencing (scRNA-seq) has emerged as a powerful tool to dissect this heterogeneity, revealing that drug target pathway activation varies significantly between primary and metastatic sites, as well as among individual cancer cells within a single site (Kim et al., 2016). For example, in metastatic renal cell carcinoma (RCC), scRNA-seq analysis of primary and lung metastatic tumors identified mutually exclusive pathway activation, leading to the design of a combinatorial regimen that co-targeted these pathways and significantly improved treatment efficacy in patient-derived xenograft models (Kim et al., 2016). Extending the dissection of heterogeneity into the realm of tumor immunity, Wang et al. (Wang et al., 2024) integrated single-cell transcriptomics with machine learning to systematically characterize intrinsic immune-evasion genes in hepatocellular carcinoma. Their study revealed that the expression of these immune-evasion genes is highly heterogeneous across distinct malignant cell subpopulations, directly dictating the differential accessibility of immunomodulatory targets at the single-cell level. This intrinsic heterogeneity in immune-evasion potential underscores the necessity of mapping not only oncogenic pathways but also immune-regulatory programs when evaluating drug target availability. Quantitative profiling of drug-target engagement at the single-cell level has further uncovered marked population heterogeneity, even at high average target engagement *in vivo* (Dubach et al., 2017). For instance, covalent BTK inhibitors and reversible PARP inhibitors showed variable target occupancy across single cells, with some cells exhibiting low occupancy despite high average levels (Dubach et al., 2017). This heterogeneity is not limited to drug binding but extends to chromatin-drug interactions: *in vivo* fluorescence lifetime imaging of doxorubicin-chromatin binding in ovarian cancer metastases revealed striking variation between different metastases in the same mouse and even between regions of the same metastasis (Sparks et al., 2018). Non-specific interactions within cells also contribute to heterogeneity, as demonstrated by slow drug diffusion kinetics in cell nuclei—three orders of magnitude slower than in vitro—leading to virtually irreversible binding to specific DNA targets (Elgart et al., 2018). These findings underscore the need for single-cell approaches to optimize drug delivery and target engagement.

The phenotypic heterogeneity of tumor cells further complicates drug target accessibility. For example, glioblastoma cells exhibit discrete distribution of integrin αvβ3, a key drug target, with surface density ranging from 0 to 1.6 molecules per μm^2^ (Wang et al., 2019). Functional studies revealed that sensitivity to integrin inhibitors correlates with target density, highlighting the importance of quantifying surface protein expression at the single-cell level (Wang et al., 2019). Similarly, single-cell analysis of renal cell carcinoma identified novel tumor-specific markers such as SPOCK1, PTGIS, and REG1A, which vary in expression across cell subpopulations and pathological types (Su et al., 2021). These markers not only serve as potential drug targets but also predict drug response, as activation of drug target pathways differs between ccRCC and type 2 pRCC (Su et al., 2021). With the integration of intracellular paired agent imaging (iPAI) and cyclic immunofluorescence (cyCIF), the TRIPODD platform allows for simultaneous measurement of single-cell drug target availability and protein expression, revealing that DTA is not always correlated with protein expression (McMahon et al., 2021). This platform provides a mechanistic understanding of therapeutic response by linking spatial context to drug efficacy, emphasizing the need for multimodal approaches to capture the full complexity of single-cell heterogeneity.

**FIGURE 1:**
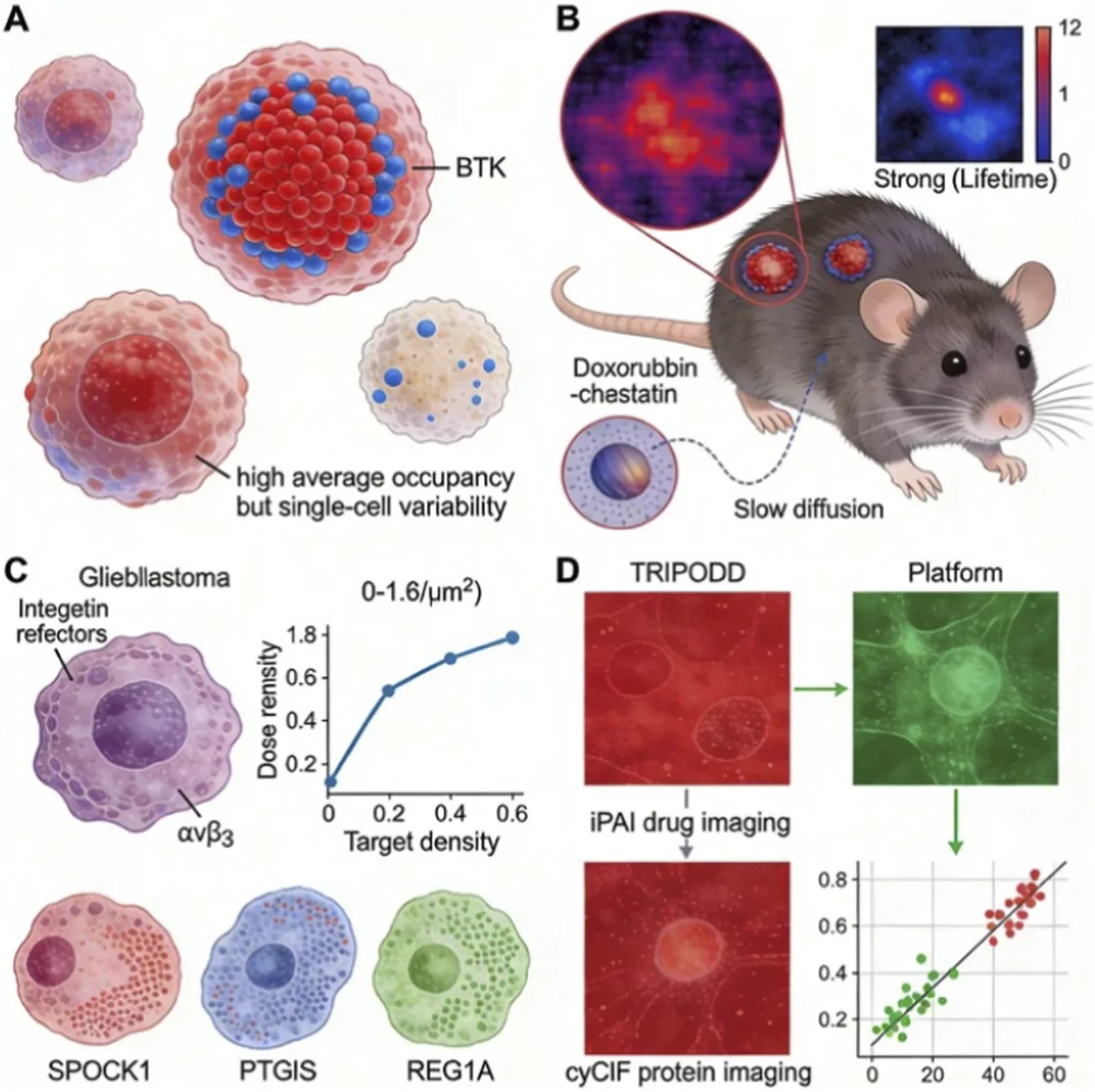
Multidimensional characteristics of single-cell tumor heterogeneity that act as critical barriers to effective cancer therapy. **(A)** Encapsulates a fundamental observation: even when a tumor cell population shows high average target occupancy (as seen with BTK inhibitors), striking single-cell variability remains, with a subset of cells maintaining low target engagement that enables treatment evasion. **(B)** Illustrates the spatial heterogeneity of *in vivo* drug-chromatin interactions, demonstrating that doxorubicin-chromatin binding differs substantially not only between distinct metastatic lesions in the same animal but also across regional subregions within a single metastasis. This panel also depicts the markedly slowed kinetics of intranuclear drug diffusion—three orders of magnitude slower than in vitro—which drives near-irreversible target binding and further amplifies intercellular differences in intracellular drug exposure. **(C)** Summarizes heterogeneous expression of cell-surface targets and tumor-specific molecular markers: in glioblastoma, integrin αvβ3 surface density ranges widely and correlates directly with cellular sensitivity to integrin inhibitors; in renal cell carcinoma, the subtype-specific markers SPOCK1, PTGIS, and REG1A are differentially expressed across cell subpopulations, contributing to divergent drug responses across pathological subtypes. **(D)** Introduces the TRIPODD multimodal imaging platform, which integrates intracellular paired-agent imaging (iPAI) for drug quantification and cyclic immunofluorescence (cyCIF) for protein profiling to simultaneously measure drug target availability and protein expression at single-cell resolution.

Taken together, the evidence outlined above demonstrates that intratumoral heterogeneity permeates every step of drug action—from target binding and subcellular distribution to phenotypic therapeutic response—and cannot be accurately captured by bulk population-level measurements alone. Figure 1 provides a consolidated visual overview of these multilayered heterogeneities and corresponding analytical approaches, framing the core challenges and technological advances in single-cell cancer pharmacology. Work using this platform has revealed an imperfect correlation between protein expression and actual drug accessibility, underscoring the limitations of single-modal protein analysis for predicting drug response. This visual synthesis reinforces the overarching conclusion that fully dissecting single-cell tumor heterogeneity demands multidimensional, spatially resolved analytical strategies. Such approaches are critical for identifying resistance-prone cell subpopulations, optimizing targeted drug delivery regimens, and advancing the clinical efficacy of precision cancer therapy.

### Signaling dynamics in cancer immunotherapy

The tumor microenvironment (TME) is a dynamic ecosystem where immune cells, stromal cells, and cancer cells communicate through complex signaling networks. Macrophages, particularly tumor-associated macrophages (TAMs), play a pivotal role in regulating innate and adaptive immune signaling (Sad et al., 2023). TAMs can exhibit either pro-tumor (M2-like) or anti-tumor (M1-like) phenotypes, with their polarization influenced by cytokines and growth factors in the TME (Sad et al., 2023). For example, cytokines such as IL-10 and TGF-β are secreted by TAMs to inhibit T cell activation and promote immune evasion (Sad et al., 2023). The PD-1/PD-L1 pathway is another critical signaling axis in cancer immunotherapy, where cancer cells upregulate PD-L1 to engage PD-1 on T cells, inhibiting their cytotoxic function (Parvez et al., 2023). Clinical trials of PD-1/PD-L1 inhibitors have shown durable responses in various cancers, but resistance remains a challenge, often due to alterations in signaling pathways or TME composition (Parvez et al., 2023).

The crosstalk between TAMs and cancer-associated fibroblasts (CAFs) further modulates signaling dynamics in the TME. CAFs secrete chemokines and growth factors that recruit and polarize TAMs toward an immunosuppressive phenotype (Timperi et al., 2024). For instance, CAFs produce CCL2, which attracts monocytes that differentiate into TAMs, while TAMs secrete TGF-β to activate CAFs, creating a positive feedback loop (Timperi et al., 2024). Single-cell RNA sequencing (scRNA-seq) has revealed that this crosstalk is spatially regulated, with specific ligand-receptor interactions occurring at the interface between TAMs and CAFs (Timperi et al., 2024). These interactions not only promote tumor growth and metastasis but also contribute to resistance to immunotherapy. For example, in pancreatic cancer, TAMs and CAFs collaborate to create a dense stromal barrier that limits T cell infiltration and drug delivery (Timperi et al., 2024). Understanding these signaling dynamics is crucial for developing combination therapies that target both cancer cells and the TME.

### Multimodal approaches in spatial pharmacology

Multimodal approaches integrate multiple data types—involving gene expression, spatial coordinates, and morphological characteristics—to provide a comprehensive view of tumor biology. The Multi-view Contrastive Graph Autoencoder (MCGAE) framework uses deep learning to combine gene expression and spatial adjacency matrices for generating unified embeddings, exceeding present methods in spatial domain identification and noise reduction (Yang et al., 2024). In the context of colorectal cancer liver metastases, MCGAE combined histological and gene expression data to locate tumor invasion zones and analyze cellular molecular regulation. (Yang et al., 2024). Similarly, the DeepFERE model fuses mass spectrometry imaging (MSI) with hematoxylin and eosin (H&E) stain microscopy to enhance spatial resolution, improving the delimitation of cancerous and para-cancerous regions (Guo et al., 2023). These multimodal imaging techniques enable the visualization of drug distribution and metabolite profiles *in situ*, providing insights into pharmacokinetics and pharmacodynamics at the tissue level.

Spatial transcriptomics (ST) has revolutionized the study of gene expression in context, with tools like mclSTExp using contrastive learning to predict spatial gene expression from H&E images (Min et al., 2024). mclSTExp integrates image features with spatial context via a Transformer encoder, achieving superior performance in predicting highly variable genes in breast cancer and skin squamous cell carcinoma datasets (Min et al., 2024). The MISO algorithm further enhances multimodal spatial omics analysis by integrating gene expression, protein expression, epigenetics, metabolomics, and histology, outperforming existing methods in identifying biologically relevant spatial domains (Coleman et al., 2025). These approaches are complemented by *in vivo* imaging techniques such as radioluminescence microscopy (RLM), which detects radiolabeled drugs at single-cell resolution, enabling the tracking of drug distribution from organs to single cells (Natarajan et al., 2015). For example, RLM revealed marked differences in rituximab binding between single cells, with some cells accumulating up to 1200 radionuclides per cell (Natarajan et al., 2015). By combining these multimodal techniques, researchers can map the spatial distribution of drugs, their targets, and immune cells, leading to more effective therapeutic strategies.

## Historical evolution of anti-cancer immunomodulators

### Development of immunomodulatory drugs in cancer treatment

The development of immunomodulatory drugs (IMiDs) has evolved from non-specific immune stimulants to targeted agents that modulate specific pathways in the immune system. Early immunotherapies included cytokines such as interferon-α and interleukin-2, which showed limited efficacy due to systemic toxicity (Mehta and Armstrong, 2016). Cancer treatment was revolutionized by the discovery of immune checkpoints, including CTLA-4 and PD-1, with ipilimumab (anti-CTLA-4) becoming the first checkpoint inhibitor approved for melanoma in 2011 (Salama et al., 2016). Later approvals of anti-PD-1/PD-L1 antibodies, including pembrolizumab and nivolumab, broadened the application of immunotherapy to different types of cancer, including non-small cell lung cancer and renal cell carcinoma (Salama et al., 2016).

Monoclonal antibodies targeting cell surface antigens have also played a key role in immunotherapy. Daratumumab, a CD38-targeting antibody, has shown significant activity in multiple myeloma by inducing complement-dependent cytotoxicity, antibody-dependent cellular cytotoxicity, and phagocytosis (van de Donk et al., 2016). Similarly, tasquinimod, an inhibitor of S100A9, targets myeloid-derived suppressor cells (MDSCs) to reduce immunosuppression in prostate cancer, with phase II trials showing prolonged progression-free survival (Mehta and Armstrong, 2016). Oral proteasome inhibitors, such as LC53-0110, have been developed to improve efficacy and safety compared to existing agents like bortezomib, with preclinical studies showing potent activity against drug-resistant multiple myeloma cells (Park et al., 2016). These advancements highlight the shift toward targeted immunomodulators that address specific immune evasion mechanisms.

### Advances in single-cell analysis techniques

The advent of single-cell analysis techniques has transformed our understanding of tumor heterogeneity and immune cell function. Early methods, such as flow cytometry, allowed for the characterization of cell surface markers, but lacked the ability to profile gene expression at the single-cell level (Cordes et al., 2023). The development of scRNA-seq in the early 2010s enabled the transcriptomic profiling of individual cells, revealing previously unrecognized cell subpopulations and their functional states (Huss et al., 2018). For example, scRNA-seq of glioblastoma identified a subpopulation of cells with high PTPN7 expression, which correlates with tumor aggressiveness and immune infiltration (Liu et al., 2025). Single-cell analysis of retinal vein occlusion (RVO) also revealed that gene expression in endothelial cells, Müller glia, and microglia was altered under ischemic stress (Feng et al., 2025).

Recent advancements in single-cell multi-omics have further expanded our capabilities, combining transcriptomics with epigenomics, proteomics, and spatial information. The annATAC tool uses a language model to annotate cell types from scATAC-seq data, outperforming other methods in accuracy (Cui et al., 2025). Spatial transcriptomics techniques, such as Visium, enable the mapping of gene expression in tissue sections, providing insights into the spatial organization of tumor cells and immune infiltrates (Su et al., 2025). For example, spatial transcriptomics of pituitary neuroendocrine tumors (PitNETs) revealed the enrichment of SPP1+ tumor-associated macrophages (TAMs) in invasive regions, which facilitate tumor invasion through the SPP1-ITGAV/ITGB1 pathway (Su et al., 2025). These techniques have not only advanced basic research but also have clinical applications, such as predicting immunotherapy response in triple-negative breast cancer (Yuan et al., 2026).

### Evolution of spatial pharmacology concepts

The concept of spatial pharmacology has evolved from traditional pharmacokinetics, which focuses on drug concentration in plasma, to a more holistic approach that considers drug distribution and target engagement within the TME. Early studies using autoradiography provided limited spatial resolution, but the development of MSI and fluorescence imaging has enabled the visualization of drug distribution at the cellular level (Natarajan et al., 2015). For example, MSI has been used to map the spatial distribution of drugs and their metabolites in tumors, revealing heterogeneous uptake and metabolism (Rajbhandari et al., 2024). The integration of spatial data with single-cell analysis has further refined our understanding of drug action, with studies showing that drug response varies not only between cell subpopulations but also based on their spatial location (Kim et al., 2016).

The evolution of spatial pharmacology is also tied to advancements in computational modeling. Multiscale modeling approaches integrate molecular, cellular, and tissue-level data to predict drug efficacy and toxicity (Clancy et al., 2016). For example, models of tumor spheroids have been used to study drug penetration and resistance, revealing that spatial gradients of oxygen and nutrients affect drug response (Ham et al., 2016). The development of 3D tumor models, such as organoids and microfluidic devices, has further enabled the study of drug action in a more physiologically relevant context (Burks et al., 2016). These models, combined with spatial imaging techniques, have provided insights into the mechanisms of drug resistance and have guided the design of more effective therapies (Dykes et al., 2016). Spatial pharmacology is an interdisciplinary field that quantitatively dissects drug spatial distribution, target engagement and pharmacodynamic outcomes in intact tissues via integrated imaging, multi-omics and multi-scale modeling, with multi-scale tissue heterogeneity as a core driver of efficacy, toxicity and resistance.

**FIGURE 2:**
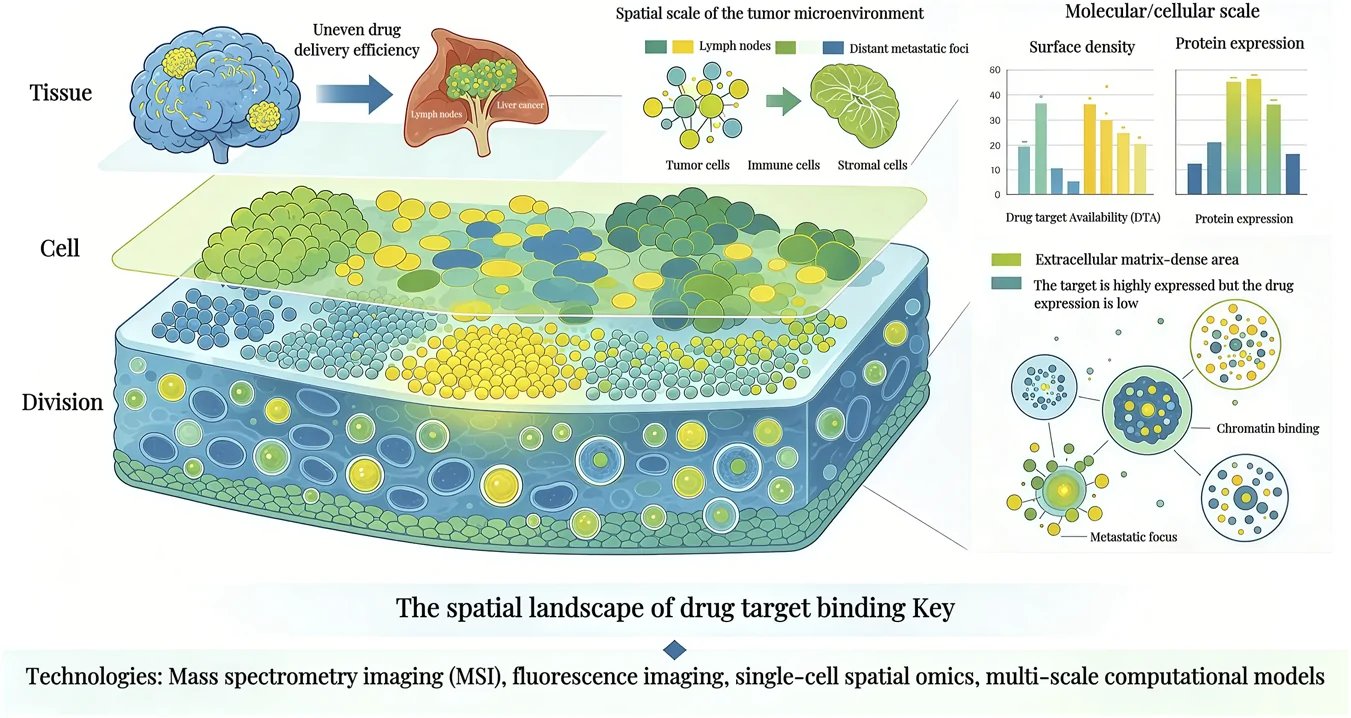
Hierarchical conceptual framework of multi-dimensional spatial heterogeneity shaping drug target engagement in the tumor microenvironment.

Figure 2 formalizes a unified conceptual model that organizes the distinct dimensions of tumor heterogeneity into a hierarchical, cascading regulatory system. In this framework, tissue-level variation in drug delivery sets the upper limit of target exposure across anatomical sites; cellular-level differences in target density and matrix accessibility define the pool of biologically available targets; and molecular-level heterogeneity in target occupancy and chromatin state determines the final magnitude of productive drug-target interactions at single-cell resolution. This multi-layered regulatory structure accounts for the complex spatial patterns of drug response that cannot be captured by traditional bulk pharmacokinetic approaches, embodying the core conceptual advancement of the spatial pharmacology paradigm.

## Current clinical practices in anti-cancer immunomodulation

### Application of spatial pharmacology in clinical settings

Spatial pharmacology is increasingly being integrated into clinical practice to optimize treatment strategies and predict patient outcomes. For example, in metastatic renal cell carcinoma, scRNA-seq has been used to identify mutually exclusive drug target pathways, leading to the design of combinatorial regimens that significantly improve efficacy (Kim et al., 2016). In a phase II study, patients with relapsed or refractory Hodgkin lymphoma treated with entinostat, a histone deacetylase inhibitor, showed a disease control rate of 24%, with tumor reduction observed in 58% of evaluable patients (Batlevi et al., 2016). Spatial pharmacology has also been applied to predict warfarin dose requirements, with the DBCSMOTE algorithm improving the accuracy of dose prediction by addressing data imbalance (Tao et al., 2020). This algorithm combines density-based spatial clustering with synthetic minority oversampling, resulting in a 47.8% accuracy in predicting doses within 20% of the actual therapeutic dose (Tao et al., 2020).

In immunotherapy, spatial pharmacology is used to monitor immune cell infiltration and drug distribution. For example, 18F-FDG PET-CT has been used to detect immune-mediated side effects of ipilimumab, such as pancreatitis and hypophysitis, by identifying abnormal FDG uptake (Wachsmann et al., 2017). Multimodal imaging, including MRI and PET, has also been used to track the delivery of cell-based therapies, such as mesenchymal stem cell (MSC) sheets, in digestive fistula models (Rahmi et al., 2016). These imaging techniques not only monitor treatment response but also provide insights into the mechanisms of action, such as the integration of MSCs into host tissue or the release of signaling molecules (Rahmi et al., 2016).

### Single-cell heterogeneity in drug response

Single-cell heterogeneity significantly impacts drug response, with studies showing that subpopulations of cancer cells can exhibit distinct sensitivity to therapies. For example, in glioblastoma, single-cell-derived subclones showed variable sensitivity to temozolomide and ionizing radiation, with some subclones being inherently resistant (Akgül et al., 2019). Similarly, in breast cancer, patient-derived organoids recapitulated the heterogeneity of the original tumor, with different subpopulations showing varying responses to oxaliplatin (Chen et al., 2018). These findings highlight the need for personalized treatment strategies that target multiple subpopulations.

The use of microfluidic devices has enabled the high-throughput screening of single-cell drug responses. For example, a hydrogel microstructure array (HMA) was used to generate CSC spheroids from single cells, revealing morphological and phenotypic variability in drug response (Afrimzon et al., 2016). The mass accumulation rate (MAR) of single cells, measured using a suspended microchannel resonator, was found to accurately predict drug sensitivity in glioblastoma and B-cell acute lymphocytic leukemia cells (Stevens et al., 2016). This method can be used with small sample sizes (25 μL of peripheral blood) while maintaining cell viability, making it suitable for clinical applications (Stevens et al., 2016).

### Signaling pathways targeted by immunomodulators

Immunomodulators target a variety of signaling pathways to enhance anti-tumor immunity. The PD-1/PD-L1 pathway is a major target, with inhibitors such as pembrolizumab and nivolumab blocking the interaction between PD-1 on T cells and PD-L1 on cancer cells (Parvez et al., 2023). These inhibitors have shown durable responses in multiple cancers, but resistance often develops due to alterations in the pathway or TME (Parvez et al., 2023). The CTLA-4 pathway is another key target, with ipilimumab blocking CTLA-4 to enhance T cell activation (Salama et al., 2016). Combination therapies targeting both PD-1 and CTLA-4 have shown improved efficacy in melanoma and renal cell carcinoma (Salama et al., 2016).

The PI3K/AKT/mTOR pathway, which is involved in cell proliferation and survival, is another signaling pathway affected by immunomodulators. Inhibitors of this pathway, such as idelalisib, have been approved for the treatment of chronic lymphocytic leukemia (CLL) (Myhrvold et al., 2018). The TGF-β pathway is also a target, as TGF-β suppresses T cell activation and promotes immune evasion. Inhibitors of TGF-β, such as galunisertib, are being evaluated in clinical trials for a range of cancers (Timperi et al., 2024). Additionally, the cGAS-STING pathway, which activates the innate immune response, is being targeted to enhance anti-tumor immunity (Lu et al., 2026). Beyond these canonical pathways, the tumor extracellular matrix actively participates in immune signaling. Lian et al. (Lian et al., 2025) identified the proteoglycan Serglycin as a critical extracellular modulator in primary liver cancer, demonstrating that it shapes an immunosuppressive tumor microenvironment by orchestrating interactions with tumor-associated macrophages. This Serglycin-macrophage axis represents a spatially confined signaling pathway, offering a novel target for immunomodulators designed to disrupt pro-tumoral communication directly within the tissue context. These pathways are often dysregulated in cancer, and targeting them can restore immune function and improve treatment outcomes.

## Diagnostic techniques in spatial pharmacology

### Single-cell sequencing for drug target accessibility

Single-cell sequencing techniques, such as scRNA-seq and scATAC-seq, are used to identify drug targets and assess their accessibility at the single-cell level. For example, scRNA-seq of metastatic renal cell carcinoma revealed variable activation of drug target pathways between primary and metastatic sites, leading to the design of combinatorial therapies (Kim et al., 2016). scATAC-seq has been used to study chromatin accessibility in prostate cancer cells treated with enzalutamide, identifying signature gene sets associated with early response and resistance (Fan et al., 2023). These studies show that chromatin closing is associated with downregulated genes during early response, while chromatin opening is linked to upregulated genes in resistant cells (Fan et al., 2023).

Single-cell sequencing is also used to profile immune cells in the TME. For example, scRNA-seq of CD8^+^ T cells in aging humans identified transcription factor networks regulating senescence, with inhibition of AP1, KLF5, or RUNX2 restoring T cell function (Turano et al., 2026). In addition, single-cell sequencing of tumor-associated macrophages (TAMs) in atherosclerosis revealed that TGFβ signaling is crucial for maintaining microglial resilience to myelin degeneration (Zhu et al., 2026). These findings highlight the potential of single-cell sequencing to identify novel drug targets and predict treatment response.

### Imaging techniques for signaling dynamics

Imaging techniques play a critical role in visualizing signaling dynamics in the TME. Fluorescent reporters, such as genetically encoded calcium indicators, enable the real-time monitoring of signaling events in live cells (Ni et al., 2018). For example, live-cell imaging of WNT signaling using fluorescent reporters has revealed the spatial and temporal regulation of this pathway in development and disease (Van der Wal and van Amerongen, 2023). Multiparametric phospho-specific flow cytometry has been used to characterize signaling network responses in B and T lymphocytes, revealing discrete alterations in network connectivity in neuropsychiatric disorders (Lago et al., 2020).

*In vivo* imaging techniques, such as intravital microscopy, provide insights into the dynamic interactions between immune cells and cancer cells. For example, a multi-modal multi-spectral intravital macroscopic imaging platform was used to monitor tumor signaling dynamics and immune cell enzyme activities concurrently (Liu et al., 2021). This platform combines bioluminescent and fluorescent reporters to track immune cell infiltration and drug distribution in real time (Liu et al., 2021). Additionally, optical coherence tomography (OCT) with plasmonic labeling has been used to track the migration of tumor-associated myeloid cells in glioblastoma models, providing high-resolution images of cell movement (Sorelle et al., 2019).

### Multimodal diagnostics in cancer immunotherapy

Multimodal diagnostics combine multiple imaging and molecular techniques to provide a comprehensive view of tumor biology. For example, the TRIPODD platform integrates iPAI and cyCIF to quantify single-cell drug target availability and protein expression, revealing that DTA is not always correlated with protein expression (McMahon et al., 2021). This platform has been used to study the heterogeneity of drug response in ovarian cancer, identifying subpopulations of cells with low target occupancy despite high average levels (McMahon et al., 2021). Another example is the combination of MSI and H&E imaging, which enhances the spatial resolution of drug distribution and improves the delimitation of cancerous regions (Guo et al., 2023).

Multimodal imaging is also used to monitor immunotherapy response. For example, 18F-FDG PET-CT has been used to detect immune-mediated side effects of ipilimumab, such as pancreatitis and hypophysitis (Wachsmann et al., 2017). Magnetic resonance imaging (MRI) and PET have been combined to track the delivery of MSC sheets in digestive fistula models, showing that the sheets integrate into host tissue and promote healing (Rahmi et al., 2016). These multimodal approaches not only improve diagnostic accuracy but also provide insights into the mechanisms of treatment response and resistance.

## Therapeutic strategies and challenges

### Overcoming single-cell heterogeneity in treatment

Single-cell heterogeneity is a major challenge in cancer treatment, as it leads to drug resistance and treatment failure. Combinatorial therapies that target multiple pathways or subpopulations have shown promise in overcoming this heterogeneity. For example, in metastatic renal cell carcinoma, scRNA-seq identified mutually exclusive pathway activation, leading to the design of a combinatorial regimen that co-targeted these pathways and significantly improved efficacy (Kim et al., 2016). In glioblastoma, single-cell-derived subclones showed variable sensitivity to targeted therapies, and combination therapy with AURK, CDK, and EGFR inhibitors effectively eliminated resistant subclones (Akgül et al., 2019).

Another strategy to overcome heterogeneity is the use of adaptive therapies, which adjust treatment based on the evolving tumor landscape. For example, in BRAF V600E-mutant cancer, simultaneously targeting RAF, MEK, and ERK kinases set a high fitness barrier, stopping the spread of subclones with significant BRAF amplification (Xue et al., 2017). In 11 out of 11 patient-derived xenografts (PDXs) of lung cancer or melanoma, this strategy proved effective without any noticeable toxicity (Xue et al., 2017). Additionally, single-cell sequencing has been used to track the evolution of drug resistance, revealing that polyclonal invasion occurs in breast tumors, with multiple clones escaping the ducts and establishing invasive carcinomas (Casasent et al., 2018).

### Enhancing drug target accessibility

Enhancing drug target accessibility is critical for improving treatment efficacy. One approach is the use of tumor-penetrating peptides, such as iRGD, which improve the penetration of nanomedicines into tumor parenchyma. For example, iRGD was used to enhance the delivery of a floxuridine (FUDR)-based nanomedicine in an orthotopic pancreatic tumor model, resulting in improved therapeutic efficacy (Li et al., 2024). Another approach is the use of targeted drug delivery systems, such as antibody-drug conjugates (ADCs), which deliver cytotoxic drugs to specific tumor cells. For example, trastuzumab emtansine (T-DM1) targets HER2-positive breast cancer cells, improving survival in patients with metastatic disease (Tsironis et al., 2018).

The tumor microenvironment (TME) also plays a role in drug accessibility, with factors such as stromal density and hypoxia limiting drug penetration. Strategies to modulate the TME, such as targeting CAFs or TAMs, can improve drug delivery. For example, losartan, an angiotensin II type 1 receptor (AT1R) blocker, has been shown to reduce stromal stiffness and improve vascular perfusion in pancreatic cancer, enhancing the delivery of chemotherapeutic agents (Wang et al., 2026). Additionally, the use of nanoparticles that respond to TME cues, such as pH or redox potential, can improve drug release at the tumor site (Jasiewic et al., 2024).

**FIGURE 3:**
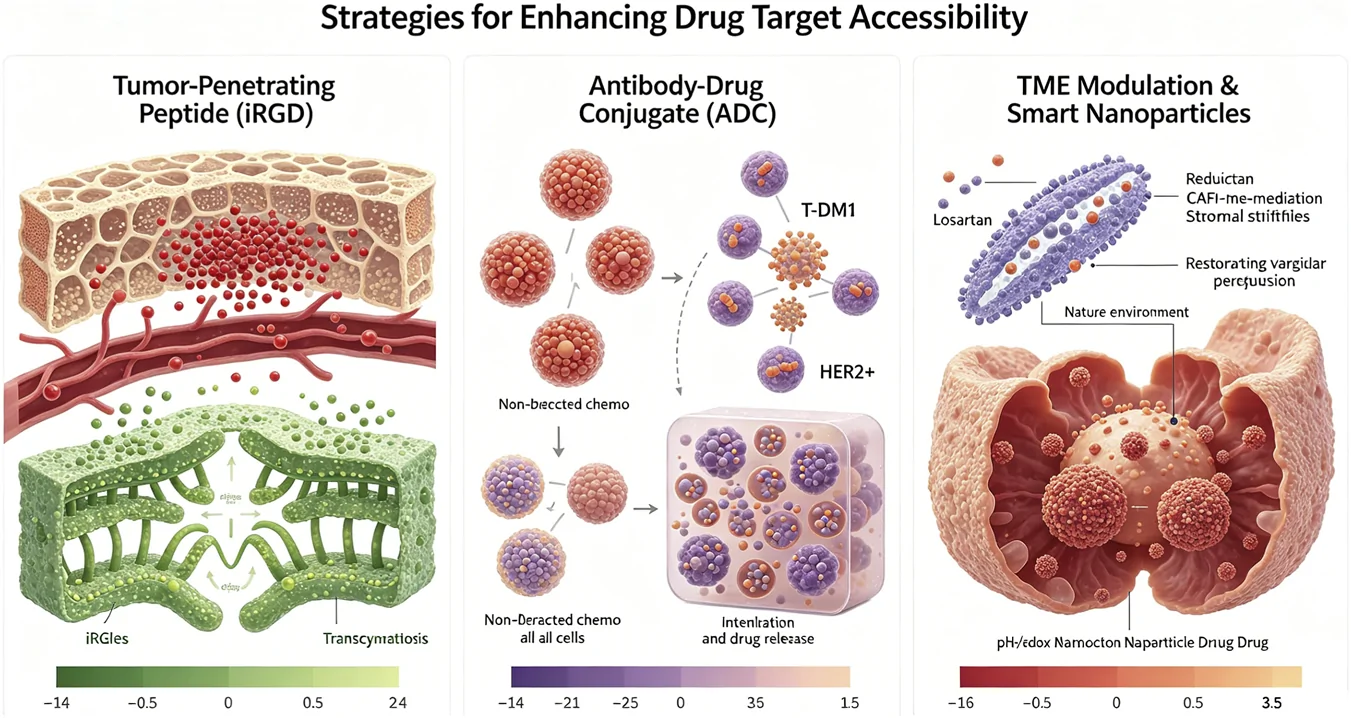
Representative strategies for enhancing drug target accessibility in cancer therapy. This three-panel schematic depicts distinct approaches to break tumor delivery barriers, with gradient heatmaps below each panel quantifying delivery performance. Left panel: iRGD peptides mediate endothelial transcytosis to overcome dense extracellular matrix and enable deep intratumoral penetration of nanomedicines. Middle panel: HER2-targeted ADC T-DM1 selectively binds and kills HER2-positive tumor cells, avoiding the non-specific toxicity of untargeted chemotherapy. Right panel: Losartan relieves CAF-induced stromal stiffness to restore tumor vascular perfusion; pH/redox-responsive smart nanoparticles further achieve tumor-specific on-site drug release in the tumor microenvironment.

In summary, the evidence presented herein illustrates that intratumoral heterogeneity influences every stage of drug action, encompassing target binding, subcellular distribution, and phenotypic therapeutic response. This complexity cannot be adequately represented by bulk population-level measurements alone. Figure 3 offers a comprehensive visual summary of these multifaceted heterogeneities and the associated analytical methodologies, highlighting the fundamental challenges and technological advancements in the field of single-cell cancer pharmacology. Work using this platform has revealed an imperfect correlation between protein expression and actual drug accessibility, underscoring the limitations of single-modal protein analysis for predicting drug response. This visual synthesis reinforces the overarching conclusion that fully dissecting single-cell tumor heterogeneity demands multidimensional, spatially resolved analytical strategies. Such techniques are important for recognizing resistance-prone cell subpopulations, fine-tuning targeted drug delivery regimens, and improving the clinical efficacy of precision cancer treatments.

### Addressing signaling dynamics in therapy

Addressing signaling dynamics in therapy requires a deep understanding of the complex interactions between cancer cells, immune cells, and the TME. One strategy is to target key signaling pathways that regulate immune evasion, such as the PD-1/PD-L1 pathway. Combination therapies that target multiple pathways, such as PD-1 and CTLA-4, have shown improved efficacy in melanoma and renal cell carcinoma (Salama et al., 2016). Another strategy is to modulate the TME to enhance anti-tumor immunity, such as using cytokines or immunomodulators to activate T cells. For example, IL-2 has been used to expand effector T cells, but its systemic toxicity limits its clinical use (Mehta and Armstrong, 2016). Beyond biochemical signaling cascades, dynamic mechanical cues within the TME also shape immune function and therapeutic resistance. Addressing these physical signaling dynamics is emerging as a Frontier. Huo et al. (2025) systematically characterized mechanosensitive ion channel-related genes in hepatocellular carcinoma and identified a prognostic gene signature that is intimately associated with both drug resistance and immune modulation. This study reveals that tumor cells actively sense and transduce mechanical forces from their surroundings to dynamically regulate their immunogenicity and susceptibility to therapy, suggesting that co-targeting mechanotransduction pathways could overcome dynamic immune escape and treatment failure driven by the physical microenvironment.

The use of adaptive cell therapies, such as CAR-T cells, has revolutionized the treatment of hematological malignancies. CAR-T cells targeting CD19 have shown high response rates in B-cell acute lymphoblastic leukemia (ALL) and diffuse large B-cell lymphoma (DLBCL) (Feins et al., 2019). However, challenges remain in solid tumors, including limited infiltration and immunosuppression in the TME (Feins et al., 2019). To address these challenges, researchers are engineering CAR-T cells to express cytokines or checkpoint inhibitors, enhancing their ability to penetrate and survive in the TME (Feins et al., 2019). Additionally, the use of bispecific antibodies, which target both tumor cells and immune cells, is being explored to redirect T cells to tumor sites (Feins et al., 2019).

## Future directions in anti-cancer immunomodulation

### Innovations in spatial pharmacology techniques

Innovations in spatial pharmacology techniques are poised to revolutionize cancer treatment. Spatial transcriptomics (ST) is evolving to provide higher resolution and multimodal integration, with tools like Xenium enabling single-cell spatial analysis of gene expression (Sakai et al., 2025). Xenium was used to study the TME after radiotherapy combined with anti-PD-L1 therapy, revealing an increase in CXCL9+ cells and CXCL13 + T cells around tumor cells (Sakai et al., 2025). Another innovation is the use of mass spectrometry imaging (MSI) to map the spatial distribution of drugs and metabolites, with recent advances in DESI and MALDI MSI providing subcellular resolution (Körber et al., 2025).

Artificial intelligence (AI) is also playing a key role in spatial pharmacology, with machine learning models being used to analyze complex spatial data. For example, the OmiCLIP model links H&E images with transcriptomics data, enabling the prediction of gene expression from histology (Chen et al., 2025). AI-driven models are also applied to discover drug-responsive subpopulations in triple-negative breast cancer, merging bulk and single-cell transcriptomics data (Wang Y. et al., 2025). These models can predict how cell populations respond to treatment, enabling the design of personalized therapies (Wang Y. et al., 2025).

### Prospects for personalized cancer immunotherapy

Personalized cancer immunotherapy is becoming a reality with the integration of single-cell analysis, spatial pharmacology, and AI. Neoantigen vaccines, which target mutation-derived antigens, are being developed for personalized treatment, with early clinical trials showing promising results (Song et al., 2018). For example, a personalized neoantigen vaccine was shown to induce T cell responses in melanoma patients, leading to tumor regression (Song et al., 2018). Another approach is the use of CAR-T cells engineered to target patient-specific antigens, with clinical trials showing efficacy in hematological malignancies (Feins et al., 2019).

Biomarkers are also critical for personalized immunotherapy, with PD-L1 expression, tumor mutation burden (TMB), and microsatellite instability (MSI) being used to predict response to checkpoint inhibitors (Sankar et al., 2022). However, these biomarkers have limitations, and researchers are exploring novel biomarkers, such as spatial immune cell infiltration and T cell receptor (TCR) clonality (Sankar et al., 2022). Extending the biomarker landscape into the realm of natural product-guided immunotherapy, Wu et al. (2026) harnessed integrative single-cell and spatial transcriptomics to decode a baicalein-responsive 10-gene signature in non-small cell lung cancer (NSCLC). Baicalein, a bioactive flavonoid from the traditional Chinese medicine Scutellaria baicalensis, has well-documented immunomodulatory properties, yet its precise cellular targets and spatially defined mechanisms of action had remained elusive. By simultaneously resolving transcriptional responses at single-cell resolution and mapping them across the tumor architecture, this study identified a spatially informed gene signature that not only predicts sensitivity to baicalein but also delineates the immune and stromal niches through which it exerts its anti-tumor effects. This work illustrates how integrative spatial multi-omics can transform a natural immunomodulatory compound into a biomarker-driven precision therapeutic, offering a paradigm for developing mechanism-based companion diagnostics for traditional medicine-derived anticancer agents. Additionally, liquid biopsies, which detect circulating tumor cells (CTCs) and cell-free DNA (cfDNA), are being used to monitor treatment response and detect minimal residual disease (Park et al., 2018).

### Emerging trends in single-cell analysis and multimodal approaches

Emerging trends in single-cell analysis include the integration of multiple omics layers, such as transcriptomics, epigenomics, and proteomics, to provide a comprehensive view of cell function (Ma et al., 2020). Single-cell multi-omics techniques, such as CITE-seq and REAP-seq, enable the simultaneous profiling of gene expression and protein levels, revealing the functional states of cells (Ma et al., 2020). Another trend is the use of spatial multi-omics, which combines spatial transcriptomics with proteomics or metabolomics to map the spatial distribution of molecules in tissues (Sharma et al., 2025).

AI-driven analysis of single-cell and spatial data is also an emerging trend, with deep learning models being used to identify cell types, predict drug response, and infer gene regulatory networks (He et al., 2023). For example, the scMODAL framework integrates single-cell multi-omics data to identify cell subpopulations and their functional states (Wang G. et al., 2025). Additionally, foundation models, such as large language models (LLMs), are being applied to single-cell data to generate hypotheses and predict cell behavior (Li et al., 2026). These trends are expected to accelerate the development of personalized cancer immunotherapy and improve patient outcomes.
